# The role of income in brain tumor patients: a descriptive register-based study: No correlation between patients’ income and development of brain cancer

**DOI:** 10.1007/s12032-018-1108-5

**Published:** 2018-03-13

**Authors:** Jonas Nilsson, Georg Holgersson, Jacob Järås, Stefan Bergström, Michael Bergqvist

**Affiliations:** 1 Center for Research and Development, Uppsala University/County Council of Gävleborg, Gävle Hospital, 801 87 Gävle, Sweden; 2 Department of Oncology, Gävle Hospital, 801 87 Gävle, Sweden; 3 Department of Radiation Sciences and Oncology, Umeå University, 901 87 Umeå, Sweden; 4 Regional Cancer Center Stockholm-Gotland, Västgötagatan 2, Box 6909, 102 39 Stockholm, Sweden; 5 Department of Radiology, Gävle Hospital, 801 87 Gävle, Sweden

**Keywords:** Brain cancer, Incidence trend, Income, Cancer Register, Socioeconomic status

## Abstract

Socioeconomic status (SES) and its association with cancer in general have been thoroughly studied in the last decades. Several studies have shown associations between SES and many types of cancer such as lung cancer, breast cancer, and prostate cancer. For gliomas, no clear occupational or exposure risk factors have been identified, although some possible risk factors such as use of cellular telephone are still controversial. The aim in the present study is to analyze whether there is an association between SES and development of brain cancer. Data from 1999 through 2013 were collected from the Swedish Cancer Registry and from the National Statistics of Sweden. Age-standardized incidence rates for people with different income were calculated using linear regression model. A total of 11,892 patients were included, of which 5675 were meningiomas, 1216 low-grade gliomas, and 5001 high-grade gliomas. No clear trend between increasing incidence rates and higher income was seen in neither of the investigated brain tumor histologies. In conclusion, the results should be interpreted with caution, but there does not seem to be a correlation in this material between increased income and development of brain cancer.

## Introduction

Primary central nervous system tumors (CNST) are classified into a four-point scale according to the 2007 WHO classification based on histopathology [1]. By clinical means, this grading system is an important tool to decide choice of therapy. Low-grade tumors (WHO I–II) are less likely to relaps, are mostly treated by surgery only, and have a good prognosis. High-grade tumors (WHO III–IV) are on the other hand regarded as malignant and normally have a poor prognosis [2].

There are numerous types of brain tumors, most emerged from the cells that support the brain cells. These cells are called glial cells and tumors consisting of glial cells are called gliomas. Meningiomas on the other hand are brain tumors that arise from the meninges of the brain and account for approximately one-third of all intracranial brain tumors in adults. Frequently, they are discovered by chance, and surgical resection is used to get histopathological diagnosis and tumor removal [3].

Socioeconomic status (SES) and its association with cancer in general have been thoroughly studied in the last decades. Several studies have shown associations between SES and many types of cancer such as lung cancer, breast cancer, and prostate cancer [4–7]. However, no clear association between SES and glioma has been identified, though the use of mobile phone and its association to gliomas is still controversial [8, 9]. This study aims to investigate whether there are any associations between SES (in terms of income) and CNST, or more specifically to high-grade gliomas (HGG), low-grade gliomas (LGG), and meningiomas.

## Materials and methods

From the Swedish Cancer Registry brain tumor cases were first identified in patients between 1980 and 2013. Classification of the tumors in the Swedish Cancer Registry during the period of study follows the *International Classification of Diseases* (*ICD*)-7. The tumors were included in the study if their *ICD*-7 codes were 193 and if their pathologic codes were either 461, 463, or 466 (meningioma), 475 (low-grade glioma, LGG), or 476 (high-grade glioma, HGG). In cases where the patients had been diagnosed with more than one brain tumor, the first tumor per diagnosis and patient were included.

In this report, direct age-standardized incidence rates have been calculated to measure trends over time. Direct age-standardization requires that the age-specific rates of the study population are known. The age-specific rates are then applied to one standard population, and here the Swedish population year 2000, i.e., the weights used, are the same for the different study populations. The age-specific rates are then summed up to the age-standardized rate. Age-standardization account for variations in the age structure of the population being looked upon as any difference in the rates over time or between geographical regions does not merely reflect the proportion of old or young people in the populations. The reason for using age-standardization when looking at cancer incidence rates is that elderly people are affected foremost.

In order to calculate age-standardized rates for people with different income, a table containing income distribution was used from Statistics Sweden. The table contains data in between 1999 and 2013, and thus cases prior to 1999 were excluded from the analysis. Similarly, people less than 20 years old were excluded from the analysis as well. The income data from Statistics Sweden are categorized into many different levels depending on the amount of income. In the analysis, all these levels were combined into four groups, 0–99, 100–199, 200–599, and 600+ tkr. Some categories had few observations, and when graphing the data, 3-year moving average was used.

In order to assess possible differences in the trend of the diagnosis during the study period, linear regression was used to fit a model to the age-standardized incidence rates.

The study was approved by the Regional Ethical Review Board in Uppsala; diary number 2015/313.

## Results

**Fig. 1:**
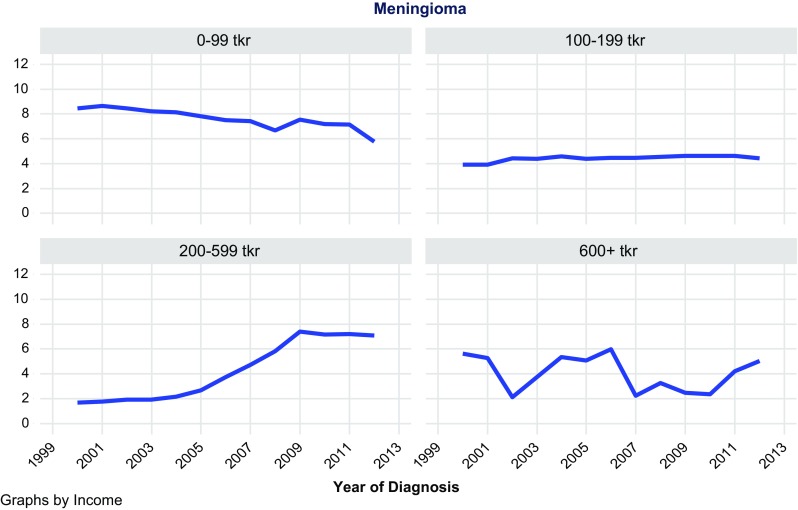
Age-standardized incidence of meningioma by income

**Fig. 2:**
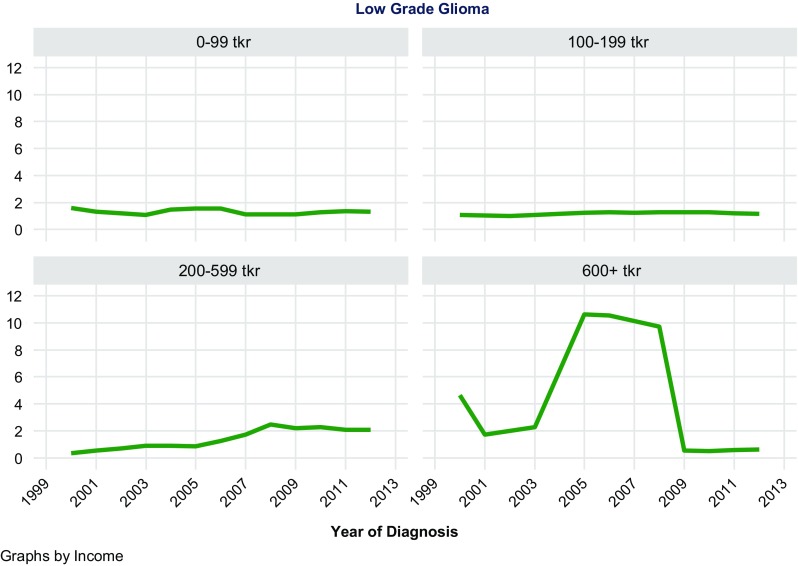
Age-standardized incidence of low grade glioma by income

**Fig. 3:**
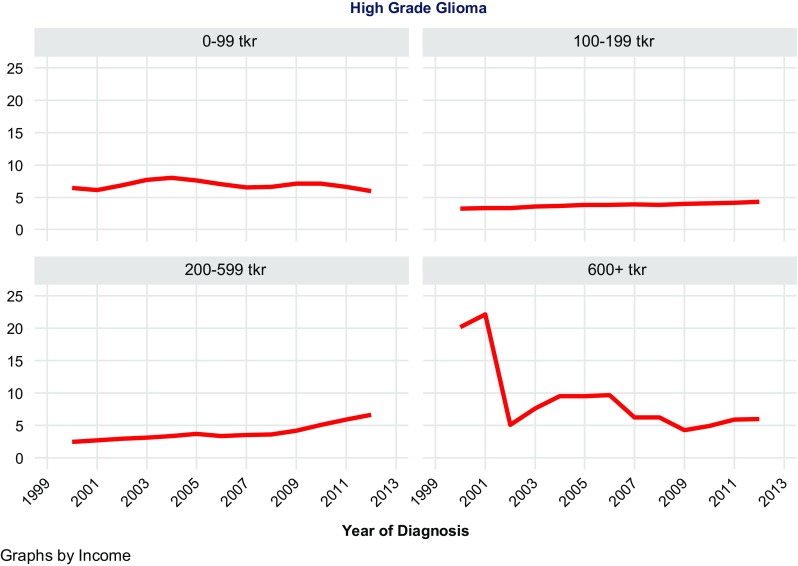
Age-standardized incidence of high grade glioma by income

A total of 11,892 patients were included in the analysis, of which 5675 were meningiomas, 1216 LGG, and 5001 HGG. In meningiomas, LGG, and HGG, the majority of patients had an income of 100–199 tkr: 4078, 923, and 3599, respectively. Very few had an income of 600 tkr or higher: 51, 20, and 119, respectively. Thus as shown in Table 1, no clear trend of incidence rate increase could be seen associated with higher SES groupings. However, as shown in Table 1 and Figs. 1, 2, 3, incidence rates of all three CNST types tended to increase up to 599 tkr and thereafter decrease.

**Table 1:** Table of regression estimates of incidence rates

Diagnosis	Group (tkr)	No. of cases	Incidence trend estimate	95% CI	*P* value
Meningioma	0–99	1253	− 0.187	− 0.248; − 0.126	< 0.001
Meningioma	100–199	4078	0.043	0.016; 0.070	0.005
Meningioma	200–599	293	0.585	0.460; 0.709	< 0.001
Meningioma	600+	51	− 0.114	− 0.368; 0.139	0.338
Low-grade glioma	0–99	179	− 0.013	− 0.043; 0.017	0.369
Low-grade glioma	100–199	923	0.016	0.003; 0.029	0.019
Low-grade glioma	200–599	94	0.178	0.128; 0.228	< 0.001
Low-grade glioma	600+	20	− 0.286	− 1.111; 0.540	0.448
High-grade gliom	0–99	987	− 0.026	− 0.128; 0.075	0.580
High-grade gliom	100–199	3599	0.081	0.070; 0.092	< 0.001
High-grade gliom	200–599	296	0.292	0.202; 0.381	< 0.001
High-grade gliom	600+tkr	119	− 1.029	− 1.720; − 0.339	0.007

## Discussion

Based on 11,892 observations of meningioma, LGG, and HGG, no general trend could be seen concerning increased income and increased risk of these CNST types. Data from these analyses must be interpreted with caution since the distribution of patients based on the above assumptions resulted in few observations in the group of high income.

Our results are in some context contradictory to the available data from the literature. It is concerning that the role of SES is known to be associated with the risk of many different cancer types, through various mechanisms [4–6]. Lung cancer is an example of a disease which is more common in smokers than in non-smokers and since low SES is associated with a higher prevalence of smoking, lung cancer is more common in low SES groups [5]. On the other hand, some cancer types such as early-stage prostate and breast cancer are discovered more in high SES groups because they often have better access to cancer screening and health care [7]. For gliomas, no clear occupational or exposure risk factors have been identified, although some possible risk factors such as cellular telephone use are still controversial [8]. Most patients with gliomas have no history of previous exposure to ionizing radiation, which is considered to be a risk factor for developing the disease [10]. There have been studies suggesting that people in certain occupations, such as physicians are at increased risk of glioblastoma, but the results from these studies have not been convincing enough for any definitive conclusions to be made [11].

The link between SES and incidence of gliomas has been previously thoroughly investigated. In an American study, data from the SEER (Surveillance, Epidemiology and End Results) Program was used to identify over 26,000 patients diagnosed with glioblastoma between 2000 and 2010 [12]. When comparing SES based on census tract of residence, it was found that higher SES was strongly associated with increased risk of glioblastoma (*p* < 0.001). Relative to patients in the lowest SES quintile, the highest SES quintile had a rate ratio of 1.45 (95% CI 1.39–1.51). In a similar study of SEER data for all glioma cases in adults > 25 years of age reported between 2000 and 2006, higher socioeconomic position based on county of residence was found to be statistically significantly associated with a higher incidence rate of glioma [13]. Patients in the highest socioeconomic position quartile had a glioma risk rate of 1.14 (95% CI 1.39–1.51) times that of the first quartile. In a study including 880 patients with glioblastoma treated at a single neurosurgical unit in the UK, socioeconomic data were obtained at ward level from government sources [14]. It was found that increasing incidence of glioblastoma was associated with increasing wage (*p* = 0.044), less unemployment (*p* = 0.0002), Indices of Multiple Deprivation (*p* = 0.05), lower population density (*p* = 0.0015), and greater ownership of cars (*p* = 0.0005). A population-based case–control study of 321 meningioma cases, 494 glioma cases, and 955 controls was carried out in Sweden between 2000–2002, and it was found that a family income in the highest quartile was associated with an increased risk of glioma (OR 1.5, 95% CI 1.1–2.1) [15]. However, socioeconomic factors were not associated with the risk of meningioma. In another case–control study by Inskip et al. of 489 glioma cases, 197 meningioma cases, 96 acoustic neurinoma cases, and 799 controls treated in three hospital in the USA between 1994 and 1998, the results showed a positive association with increasing household income for the risk of low-grade glioma, meningioma, and acoustic neurinoma but not for high-grade glioma [16]. Similarly, positive associations were observed with level of education for low-grade glioma and acoustic neuroma, but not for high-grade glioma or meningioma. In a separate study, patients were interviewed regarding their use of handheld cellular phones [17]. As compared with patients who had never, or very rarely, used a cellular telephone, the relative risks associated with a cumulative use of a cellular telephone for more than 100 h was not significantly elevated for any of the tumor entities. Neither did tumors occur disproportionately often on the side of head on which the telephone was typically used. These results are in line with the results from the large multinational INTERPHONE case–control study that included 2708 gliomas, 2409 meningiomas, and matched controls from 13 countries which showed no increase in risk of glioma or meningioma with use of mobile phones [18]. One hypothesis regarding the increased glioblastoma risk in persons with high SES is related to cellular telephone use. Before the almost universal use of cellular telephones seen nowadays, the first users of the technology in the 1980s tended to be people who could afford buying a cellular phone, that is, people of high SES levels. However, the results from several large well-designed studies such as INTERPHONE [18] contradicts this explanation, and it seems that the reason for higher incidence of gliomas in high SES groups is to be found elsewhere.

In conclusion, the results should be interpreted with caution, but there does not seem to be a correlation in this material between increased income and development of meningiomas, nor LGG or HGG.
